# Transient extracellular application of gold nanostars increases hippocampal neuronal activity

**DOI:** 10.1186/s12951-014-0031-y

**Published:** 2014-08-20

**Authors:** Kirstie Salinas, Zurab Kereselidze, Frank DeLuna, Xomalin G Peralta, Fidel Santamaria

**Affiliations:** 1 UTSA Neurosciences Institute, The University of Texas at San Antonio, San Antonio 78249, Texas, USA; 2 Department of Physics and Astronomy, The University of Texas at San Antonio, San Antonio 78249, Texas, USA

**Keywords:** Nanoparticle, Uptake, Nanotoxicity, Neurons, Potassium channels, Firing rate

## Abstract

**Background:**

With the increased use of nanoparticles in biomedical applications there is a growing need to understand the effects that nanoparticles may have on cell function. Identifying these effects and understanding the mechanism through which nanoparticles interfere with the normal functioning of a cell is necessary for any therapeutic or diagnostic application. The aim of this study is to evaluate if gold nanoparticles can affect the normal function of neurons, namely their activity and coding properties.

**Results:**

We synthesized star shaped gold nanoparticles of 180 nm average size. We applied the nanoparticles to acute mouse hippocampal slices while recording the action potentials from single neurons in the CA3 region. Our results show that CA3 hippocampal neurons increase their firing rate by 17% after the application of gold nanostars. The increase in excitability lasted for as much as 50 minutes after a transient 5 min application of the nanoparticles. Further analyses of the action potential shape and computational modeling suggest that nanoparticles block potassium channels responsible for the repolarization of the action potentials, thus allowing the cell to increase its firing rate.

**Conclusions:**

Our results show that gold nanoparticles can affect the coding properties of neurons by modifying their excitability.

## Background

Several types of nanoparticles, particularly gold, can bind to proteins on the surface of cells [1],[2]. Therefore, it is important to determine the effects that such binding has, not only on the metabolism of cells, but also, on their function [3],[4]. Although, there is a large body of work on the fatal toxic effects of gold nanoparticles on neurons there is little understanding how these widely used nanoparticles might affect their function, namely their activity and coding properties [5].

We hypothesized that gold nanoparticle-protein interactions alter the electrophysiological properties of neurons, which are mediated by proteins within the neuronal membrane. To test this hypothesis we synthesized gold nanostars using a silver-seed mediated method we recently developed [6]. We applied the nanoparticles to mouse hippocampal slices while recording the action potential activity of neurons in the CA3 area. Our results show that the firing rate of action potentials of these cells increases by 17% after nanoparticle application. The increase in activity persists after a short nanoparticle application (5 min). The shape of the action potential changes in the area associated with potassium currents, suggesting a preferential effect of these nanoparticles on potassium channels. Overall, our results show that short transient applications of gold nanoparticles have nontoxic functional effects on neurons that should be considered when developing nanotechnology for neurobiology applications.

## Results and discussion

**Figure 1:**
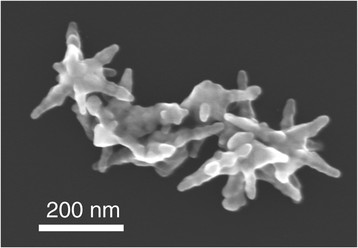
Scanning electron microscope image of gold nanostars used in this study (imaged using a Hitachi S-5500).

**Figure 2:**
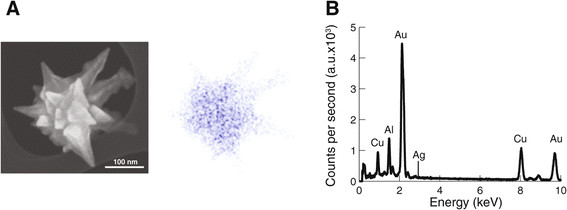
**EDS analysis shows that nanostars are made of gold. (A)** Left: SEM image of gold nanostar. Right: EDS mapping of gold atoms for nanoparticle in left. **(B)** EDS spectrum showing no silver on the surface of the nanoparticles.

**Figure 3:**
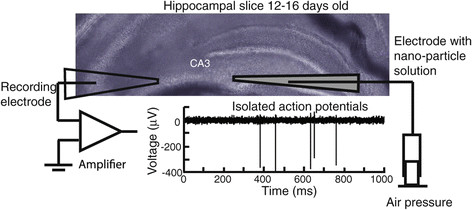
**Experimental setup used to record and deliver star shaped gold nanoparticles to the CA3 region of mouse hippocampal slices.** Transmitted light image obtained using an Olympus BX61WI microscope with a 20× 0.95 N.A. objective.

We started our work by synthetizing gold nanoparticles as described in our previous publications [6]. Our method results in star shapes of 180 nm in average width and 70% yield (Figure 1). Energy dispersive X-Ray spectroscopy (EDS) analysis confirms that the nanoparticles are made out of gold (Figure 2). As described in Methods we prepared acute hippocampal slices. A recording electrode was brought in close proximity to the CA3 area of the slice. This section contains the cell bodies of excitatory hippocampal pyramidal cells. A second electrode was also brought close to the first electrode. This electrode could contain regular artificial cerebrospinal fluid (aCSF) or nanoparticle solution (Figure 3).

**Figure 4:**
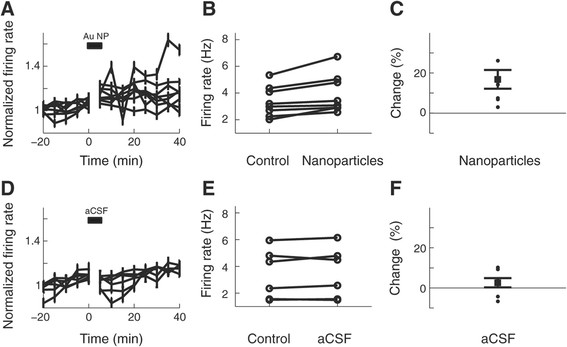
**Transient application of gold nanoparticles increases hippocampal neuronal activity. (A)** Firing rate average from extracellularly recorded CA3 hippocampal neurons before and after gold nanoparticle (Au NP) application. The activity was averaged every 5 min. Bars are S.E.M. Firing rates were normalized to values before application. **(B)** Absolute firing rate of all the experiments in (A) before and after nanoparticle application. Firing rate after application was averaged from t = 20 to t = 50 min. **(C)** Percentage change of firing rate application from B. Error bars are for the S.E.M. **(D-F)** Identical analysis as in A-C when the application pipette only contained artificial cerebrospinal fluid (aCSF) and no nanoparticles.

We recorded the firing rate activity of isolated hippocampal CA3 neurons before and after applying gold nanoparticles (Figure 4A). Our results show a consistent increase in the firing rate of the neurons after nanoparticle application (Figure 4B). Note that nanoparticles were delivered over a period of 5 min with continuous perfusion. Thus, nanoparticles that did not attach to neurons were washed out. Using a fluorescence correlation spectroscopy (FCS) setup we determined that the concentration of the nanoparticles in the pipette was about 3 nM (see Materials and methods and Additional file 1). After being released into the chamber the concentration decays as the nanoparticles diffuse and are carried by the circulating bath and enter the slice tissue, thus, it is expected that a much lower concentration of nanoparticles reaches the cells. The firing rate was averaged for 20 to 50 minutes after nanoparticle application and resulted in an average increase in firing rate of 16.82% ± 0.05 (S.E.M., p < 0.05, n = 8 experiments, Figure 4C). An identical experiment with a solution free of nanoparticles did not result in a significant increase in firing rate (2.60% ± 0.02 S.E.M, not significant, n = 6 experiments, Figure 4D-F). Similarly, the application of 100 nm diameter latex nano-beads did not cause a significant change in firing rate (2.29% ± 0.03 S.E.M., not significant, n = 3 experiments, not shown). Therefore, acute nanoparticle application on neurons results in an increase in firing rate.

**Figure 5:**
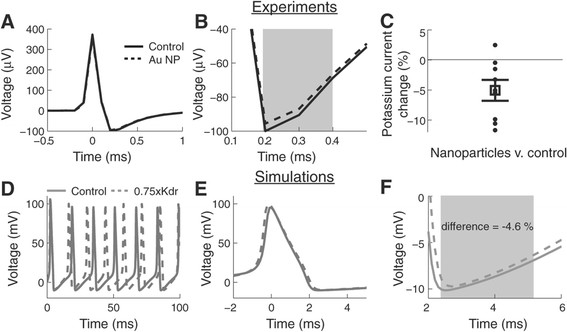
**Transient application of gold nanoparticles reduces the potassium associated current in CA3 hippocampal neurons. (A)** Average action potential before (control) and after application of gold nanoparticles (Au NP). The overlay at this scale makes the traces indistinguishable. **(B)** The hyperpolarization region of the action potential (from A) is associated with potassium currents. **(C)** The percent difference in the shaded area integrated from B before and after application of gold nanoparticles for all the experiments (5.0% ± 1.8 S.E.M., n = 8 experiments). **(D-F)** Computer simulation of action potential generation using the Hodgkin and Huxley model. **(D)** The model generated sustained firing rates when stimulated with continuous 7 nA. Varying the density of potassium currents (Kdr) by 0.75 results in an increase in firing rate of 17.5%. **(E)** Action potential overlay comparing the control and 0.75xKdr simulations. **(F)** Integrating the potassium associated voltage in the model shows a decrease in this current of 4.6%. The shaded area in F covers the same fraction of the action potential as in B.

In order to elucidate the mechanism through which nanoparticle application affected firing rates we investigated whether there was a change in the shape of the action potentials. For this reason we analyzed the average action potential shape before and after nanoparticle application (Figure 5A). We did not find changes in spike height or spike width. The action potentials rapidly repolarized after about 1.0 ms. However, the current associated with potassium channels appeared smaller than in the action potentials before nanoparticle application (Figure 5B). To quantify differences in the potassium associated current we first determined the time of the minimum voltage deflection in each action potential. The minimum voltage after the action potential peak is the time of maximum potassium current activation [7]. Since the amplitude of the potassium associated current could be affected by noise we decided to integrate the area of this section of the action potentials. Starting from the minimum voltage (maximum potassium activation) we integrated the area of the voltage trace for 0.2 ms, which corresponded to about 30% of the repolarization period (shaded area Figure 5B). We quantified this value in all the experiments before and after the application of nanoparticles. Our results show that there is a significant decrease of 5.0% ± 1.8 S.E.M (p < 0.05) in this current (Figure 5C). Thus, our data suggests that our gold nanoparticles preferentially affect potassium channels.

In order to test whether blocking of potassium channels could result in an increase of the firing rate of the recorded neurons, we implemented a computer model using the Hodgkin and Huxley equations (see Materials and methods). We varied the density of non-linear potassium channels (Kdr) while delivering a constant input current (7 nA) until we obtained the change in firing rate observed in the experiments. Our modeling results suggest that it is necessary to change the Kdr by 0.75 (from 36 mS/cm^2^ to 27 mS/cm^2^) to obtain an increase of 17.5% in the firing rate (Figure 5D). We then compared the action potential shape from the control and 0.75xKdr simulations (Figure 5E). As in the experimental results the action potential shapes were very similar to each other. However, as in the experiments there was a difference in the strength of the potassium related potential. Since the shape and firing rate of the Hodgkin and Huxley model differ from the recorded action potentials we integrated the same fraction of the voltage trace as in the experiments (30% of the repolarization time after the minimum voltage). Interestingly, our results show that the integral of the potassium voltage is reduced by 4.6% (Figure 5F). Thus, our simulations corroborate our experimental results that blocking of potassium channels by transient application of gold nanoparticles results in increased firing rates for prolonged periods of time.

## Conclusion

There is increasing evidence that nanoparticles made of different materials and shapes can be fatally toxic to the brain [8]-[10]. However, little is known about the non-fatal effects on the electrical activity of neurons. We found that a transient extracellular application of gold star-shaped nanoparticles increases the mean firing rate of CA3 hippocampal neurons. A very recent article [11] shows that intracellular injection of gold nanoparticles in the CA1 pyramidal neurons in the hippocampus results in an increase in the excitability of these cells. Our results are consistent with this report and further contribute to suggest that the site of action of the gold nanoparticles is on potassium channels. In our case, given that the reported uptake of gold nanoparticles takes much longer [12] than the effects that we measured, we hypothesize that the nanoparticles are mostly on the surface of the cells. Nanoparticles binding to potassium channels could affect their function by modifying their conformational state through adsorption [13], direct blocking [14] or clustering channels by cross-linking [15].

Our combined experimental and modeling approach suggests that a fraction of potassium channels are rapidly blocked by gold nanoparticles. This blockage causes a faster repolarization of the cell and a subsequent increase in firing rate. It is not known whether this increase in firing rate could cause pathological conditions that involve the hippocampus, such as epilepsy [16]. Increases in neuronal activity in isolated preparations could be compensated by network dynamics [17]. Oscillations in the brain are common [18]; therefore, nanoparticle induced increases in firing rate could occur without a behavioral effect. In any case, it is important to study the neurological and behavioral effects of application of gold nanoparticles in vivo [19].

In our experiments we only used a single concentration (about 3 nM) of the gold nanoparticle solution. This concentration was further decreased when combining the nanoparticle solution with aCSF and releasing it into the space of the chamber. Other factors affect the nanoparticle concentration such as the distance of the nanoparticle application electrode from the slice, the depth of the recorded neuron in the tissue, the movement of the solution in the perfusion chamber, and the diameter of the pipette tip. The uncertainty in controlling the concentration of nanoparticles in the bath did not allow us to perform concentration dependent experiments. A potential future direction to solve this problem could be to integrate our FCS measuring setup with our nanoparticle experiments to determine their concentration in each experimental condition.

Overall, our work shows that transient exposure of neurons to gold nanoparticles can affect the coding properties of the hippocampus. Thus, these effects have to be taken into account when developing nanomaterials and nanotechnology to study brain function [20].

## Materials and methods

We synthesized gold star-shaped nanoparticles using a method we recently published [6]. Briefly, silver seeds are used as a nucleating agent, upon which growth of the nanoparticle occurred. Our method produced gold nanostars at a 70% yield and a concentration of 2–4 nM. Energy dispersive X-Ray spectroscopy (EDS) was performed with a JEOL JEM-ARM200F (JEOL, JP) to determine the chemical content and relative density of individual nanoparticles. Scanning electron microscopy images were collected with this same microscope or with a Hitachi S-5500.

In order to determine the concentration of nanoparticles we modified a two-photon microscope (Prairie Technologies, Madison, WI) to perform FCS measurements. A photo-multiplier detector was removed and in its place we aligned an optical fiber coupled with a lens. The other end of the optical fiber was connected to an avalanche photo diode (Perkin Elmer, USA). The photo diode was then coupled to an auto-correlator card (Correlator.com, Hong Kong) which was connected to an acquisition computer. A typical experiment collected 10 trials for 20 seconds. Nanoparticle luminescence [21],[22] was stimulated with a femtosecond laser Chameleon (Coherent, Santa Clara, CA), at 90 MHz repetition rate with a <150 fs pulse at a wavelength of 760 nm. The luminescence acquisition dichroic had a band pass filter from 584 to 630 nm.

The general form of the auto-correlation function is:

(1) $$G\left(t\right)=\frac{1}{V_{\mathrm{eff}}\left[C\right]}\frac{1}{1+\frac{t}{t_{d}}}\frac{1}{\sqrt{1+{\left(\frac{r_{o}}{z_{o}}\right)}^{2}\frac{t}{t_{d}}}}$$

where *t* is time, *t*_*d*_ is the auto-correlation time constant, *r*_*o*_ is the waist at the focal point, *z*_*o*_ is the spread along the z-axis, *C* is the concentration and the effective volume (*V*_*eff*_) is the two-photon illumination spot

(2) Veff=π32ro2zo.

We determined the two-photon imaging volume using fluorescent beads of 0.1 μm in diameter (Invitrogen, USA). The measured waist of the focal point is *r*_*o*_ = 1.25 μm and the spread along the z-axis is *z*_*o*_ = 5.40 μm.

The auto-correlation time constant is related to the diffusion coefficient (*D*) of the particles by:

(3) $$t_{d}=\frac{{r_{o}}^{2}}{8D}\cdot$$

Finally, the amplitude at zero lag (*G*(0)) is inversely proportional to the average number of particles in the volume (*N*)

(4) $$G\left(0\right)=\frac{1}{<N>}=\frac{1}{V_{\mathrm{eff}}\left[C\right]}$$

from which we can calculate the concentration using equation (2). Fitting equation (1) to our measurements (Additional file 1: Figure S1) shows that the concentration of gold nanoparticles in our solution is 3.44 ± 0.01 nM (95% confidence intervals).

We compared the FCS concentration measurements to an estimate based on the content of gold in the formulation and the area and volume of the nanoparticles. We estimated the surface area (*A*_*S*_) and volume (*V*_*S*_) of the nanostars by measuring the radius of the core (*r*_*c*_), the length of the rays (*r*_*h*_), the radius of the base of the ray (*r*_*b*_) and estimating the overlap between a spherical cone and a sphere given by the overlapping length (Δ*r*) as

(5) $$A_{S}=4\pi {r_{c}}^{2}+\mathrm{n\pi}r_{b}\left(r_{b}+{\left({r_{b}}^{2}+{r_{h}}^{2}\right)}^{\frac{1}{2}}\right)-2\mathrm{n\pi}r_{b}\mathrm{\Delta r}$$

(6) $$V_{S}=\frac{4}{3}\pi {r_{c}}^{3}+\frac{1}{3}\mathrm{n\pi}{r_{b}}^{2}r_{h}-\frac{1}{3}\;\mathrm{n\pi \Delta r}\left(3{r_{b}}^{2}+\Delta r^{2}\right)$$

Where

(7) $$\mathrm{\Delta r}=r_{c}-{\left({r_{c}}^{2}-{r_{b}}^{2}\right)}^{1/2}$$

and *n* denotes the number of rays. From the SEM images, we found that the nanostars had an *r*_*c*_ = 36 ± 3 nm, *r*_*h*_ = 55 ± 6 nm, *n* = 5.7 ± 1.3 peaks with a range of 4–7 peaks, and *r*_*b*_ = 11 ± 1 nm. We calculated the predicted concentration given our synthesis protocol from

(8) $$C=\frac{1}{2}\frac{M_{\mathrm{Au}}\cdot V_{\mathrm{Au}}}{V_{\mathrm{nps}}\cdot N_{x}}$$

where *M*_*Au*_ is the molarity of the gold chloride solution (0.25 mM), *V*_*Au*_ is the volume of the gold chloride solution (20 mL), *V*_*nps*_ is the volume of the nanoparticle solution (3 mL) and *N*_*x*_ is the number of gold atoms in each shape (*N*_*V*_ = *V*_*R*_/*d*_*Au*_^3^ for solid stars and *N*_*A*_ = *A*_*R*_/*d*_*Au*_^2^ for hollow stars). The 1/2 comes from diluting the final solution in half. We assumed that the nanoparticles had an FCC crystalline structure and used the lattice constant of gold *d*_*Au*_ = 0.408 nm to find the number of atoms in each shape. Using this approach we predicted a concentration of stars of 0.12 nM, for solid nanoparticles, and 2.56 nM, for hollow structures (in both cases assuming *n* = 5 peaks). Therefore, the concentration based on a hollow nanoparticle calculation is in very good agreement with the concentration extracted from our experimental FCS measurements.

Artificial cerebrospinal fluid (aCSF) was composed of (in mM): NaCl, 125; KCl, 2.5; CaCl_2_, 2; MgCl_2_, 1.3; NaH_2_PO_4_, 1.25; NaHCO_3_, 26; D-glucose, 20 (Fischer Scientific, USA). C57/BL6NJ mice 14–21 day old were euthanized following a protocol approved by the IACUC of The University of Texas at San Antonio. The brain of the animals was quickly removed and was sectioned in 200 μm thick slices. After incubation for 35 minutes in 37 C the slices were transferred to a chamber and bathed in oxygenated aCSF throughout the experiments [23],[24]. The extracellular solution circulated through the chamber at about 2 mL/min.

We fabricated recording electrodes from glass capillary tubes (1–3 MΩ). Electrodes were filled with 0.2 μL of 5 M NaCl. Recordings were obtained using an Alembic VE2 amplifier (Alembic Instruments; Montreal, Canada) together with Axon pclamp software (Molecular Devices; Sunnyvale, CA). The signal was filtered between 1–2 kHz to isolate action potential waveforms and sampled at 10 kHz. Action potentials were recorded and isolated using a combination of voltage thresholds. The data was then imported into Matlab (Natick, MA) to be further analyzed.

Nanoparticles were delivered by pressure injection through a second electrode connected to a micro syringe pump (Harvard Apparatus, Cambridge, MA). This second electrode was placed near the first electrode with a micromanipulator. Approximately 1 microL (μL) of gold nanostars was added during a 5 min window. In other experiments we applied 100 nm diameter latex nanobeads (TetraSpeck nanospheres, Invitrogen).

We also implemented a standard Hodgkin and Huxley model [7]. This model computes the membrane voltage of a neuron. The full model is as follows:

(9) $$C\frac{\mathrm{dV}}{\mathrm{dt}}=-\left({\bar{g}}_{\mathrm{Na}}m^{3}h\left(V-E_{\mathrm{Na}}\right)+{\bar{g}}_{k}n^{4}\left(V-E_{k}\right)+{\bar{g}}_{\mathrm{rest}}\left(V-E_{\mathrm{rest}}\right)\right)+I$$

The passive parameters of the model are the membrane capacitance per unit area (*C* = 1 μF/cm^2^); the leak resistance $\left({\bar{g}}_{\mathrm{rest}}=0.3\;\mathrm{mS}/{\mathrm{cm}}^{2}\right)$; and the resting potential (*E*_*rest*_ = 10.6 mV). The active properties of the model consist of non-linear sodium ($\mathrm{Na}={\bar{g}}_{\mathrm{Na}}m^{3}h\left(V-E_{\mathrm{Na}}\right)$) and potassium ($K_{\mathrm{dr}}={\bar{g}}_{k}n^{4}\left(V-E_{k}\right)$) channels. The density of the Na current is ${\bar{g}}_{\mathrm{Na}}=$ 120 mS/cm^2^ and ${\bar{g}}_{K}=$ 36 mS/cm^2^ for the Kdr current. The reversal potential for potassium current is *E*_*K*_ = −12 mV and *E*_*Na*_ = 115 mV for the Na current. The activation of each current is given by a set of mass action equations described by the state variables *m* and *h* for the Na current, and *n* for the Kdr current. The value of *m*, *n*, and *h* is determined by solving the following equation for each one of them (*x* = *m*, *n*, or *h*):

(10) $$\frac{\mathrm{dx}}{\mathrm{dt}}=\alpha_{x}\left(1-x\right)-\beta_{x}x$$

The reaction rate *α*_*x*_ is called the forward reaction, and *β*_*x*_ is the backwards reaction. The forward-backward reaction rate for the activation variables are:

(11) αm=2.5−0.1Ve2.5−0.1v−1βm=4e−V18αh=0.07e−V20αn=0.1−0.01Ve1−0.1V−1αm=0.125e−V80

where *V* is the voltage from the main equation.

The input to the model was a constant current applied at *t* = 50 ms. The stimulation caused the model to depolarize and generate action potentials continuously. The model was implemented in Matlab and integrated using the Runge–Kutta algorithm.

## Abbreviations

aCSF: Artificial cerebrospinal fluid

Kdr: Non-linear potassium channels

## Competing interests

The authors declare that they have no competing interests.

## Authors’ contributions

KS participated in the experimental design and carried out the experiments. ZK synthesized the nanoparticles and oversaw their use. FD performed the control latex beads experiments. FS and XGP conceived of the study. XGP participated in the coordination of the project and helped draft the manuscript. FS participated in the design of the project, performed the analysis and drafted the manuscript. All authors read and approved the final manuscript.

## Additional file

## Supplementary Material

Additional file 1: **“Analysis of fluorescence correlation spectroscopy (FCS) measurements of star shaped nanoparticles”.** The figure contains two panels showing FCS measurements to determine the concentration of gold nanoparticles in solution and a comparison with theoretical calculations. Click here for file

## References

[B1] Chithrani BD Ghazani AA Chan WCW Determining the size and shape dependence of gold nanoparticle uptake into mammalian cells Nano Lett 2006 6 662 668 10.1021/nl052396o 16608261

[B2] Lin C-C Yeh Y-C Yang C-Y Chen C-L Chen G-F Chen C-C Wu Y-C Selective binding of mannose-encapsulated gold nanoparticles to type 1 pili in escherichia coli J Am Chem Soc 2002 124 3508 3509 10.1021/ja0200903 11929231

[B3] Connor EE Mwamuka J Gole A Murphy CJ Wyatt MD Gold nanoparticles are taken up by human cells but do not cause acute cytotoxicity Small 2005 1 325 327 10.1002/smll.200400093 17193451

[B4] Alkilany A Murphy C Toxicity and cellular uptake of gold nanoparticles: what we have learned so far? J Nanoparticle Res 2010 12 2313 2333 10.1007/s11051-010-9911-8 PMC2988217 21170131

[B5] Yang Z Liu ZW Allaker RP Reip P Oxford J Ahmad Z Ren G A review of nanoparticle functionality and toxicity on the central nervous system J R Soc Interface 2010 7 S411 S422 10.1098/rsif.2010.0158.focus 20519209 PMC2943893

[B6] Kereselidze Z, Romero VH, Peralta XG, Santamaria F: **Gold nanostar synthesis with a silver seed mediated growth method.** *J Vis Exp* 2012, **ᅟ:**ᅟ. 10.3791/3570 PMC3462575 22297908

[B7] Koch C Biophysics of computation : information processing in single neurons 1999 Oxford University Press, New York

[B8] de Oliveira GM Kist LW Pereira TC Bortolotto JW Paquete FL de Oliveira EM Leite CE Bonan CD de Souza Basso NR Papaleo RM Bogo MR Transient modulation of acetylcholinesterase activity caused by exposure to dextran-coated iron oxide nanoparticles in brain of adult zebrafish Comp Biochem Physiol C Toxicol Pharmacol 2014 162 77 84 10.1016/j.cbpc.2014.03.010 24704546

[B9] Knudsen KB Northeved H Ek PK Permin A Andresen TL Larsen S Wegener KM Lam HR Lykkesfeldt J Differential toxicological response to positively and negatively charged nanoparticles in the rat brain Nanotoxicology 2014 8 764 774 23889261 10.3109/17435390.2013.829589

[B10] Sharma A Muresanu DF Patnaik R Sharma HS Size- and age-dependent neurotoxicity of engineered metal nanoparticles in rats Mol Neurobiol 2013 48 386 396 10.1007/s12035-013-8500-0 23821031

[B11] Jung S Bang M Kim BS Lee S Kotov NA Kim B Jeon D Intracellular gold nanoparticles increase neuronal excitability and aggravate seizure activity in the mouse brain PLoS One 2014 9 e91360 10.1371/journal.pone.0091360 24625829 PMC3953378

[B12] Chithrani BD Chan WCW Elucidating the mechanism of cellular uptake and removal of protein-coated gold nanoparticles of different sizes and shapes Nano Lett 2007 7 1542 1550 10.1021/nl070363y 17465586

[B13] Pan H Qin M Meng W Cao Y Wang W How do proteins unfold upon adsorption on nanoparticle surfaces? Langmuir 2012 28 12779 12787 10.1021/la302258k 22913793

[B14] Park KH Chhowalla M Iqbal Z Sesti F Single-walled carbon nanotubes are a new class of ion channel blockers J Biol Chem 2003 278 50212 50216 10.1074/jbc.M310216200 14522977

[B15] Verma A Stellacci F Effect of surface properties on nanoparticle–cell interactions Small 2010 6 12 21 10.1002/smll.200901158 19844908

[B16] Ribeiro FM Paquet M Cregan SP Ferguson SSG Group I metabotropic glutamate receptor signalling and its implication in neurological disease CNS Neurol Disord Drug Targets 2010 9 574 595 10.2174/187152710793361612 20632969

[B17] Yang F, Liu ZR, Chen J, Zhang SJ, Quan QY, Huang YG, Jiang W: **Roles of astrocytes and microglia in seizure-induced aberrant neurogenesis in the hippocampus of adult rats.** *J Neurosci Res* 2009,. 10.1002/jnr.22224 19774666

[B18] Raghavachari S Lisman JE Tully M Madsen JR Bromfield EB Kahana MJ Theta oscillations in human cortex during a working-memory task: evidence for local generators J Neurophysiol 2006 95 1630 1638 10.1152/jn.00409.2005 16207788

[B19] Sriramoju B, Kanwar RK, Kanwar JR: **Nanomedicine based nanoparticles for neurological disorders.** *Curr Med Chem* 2014, **ᅟ:**ᅟ. Epub ahead of print. 10.2174/0929867321666140716095644 25039778

[B20] Alivisatos AP Andrews AM Boyden ES Chun M Church GM Deisseroth K Donoghue JP Fraser SE Lippincott-Schwartz J Looger LL Masmanidis S McEuen PL Nurmikko AV Park H Peterka DS Reid C Roukes ML Scherer A Schnitzer M Sejnowski TJ Shepard KL Tsao D Turrigiano G Weiss PS Xu C Yuste R Zhuang X Nanotools for neuroscience and brain activity mapping ACS Nano 2013 7 1850 1866 10.1021/nn4012847 23514423 PMC3665747

[B21] Farrer RA Butterfield FL Chen VW Fourkas JT Highly efficient multiphoton-absorption-induced luminescence from gold nanoparticles Nano Lett 2005 5 1139 1142 10.1021/nl050687r 15943457

[B22] Wang H Huff TB Zweifel DA He W Low PS Wei A Cheng J-X In vitro and in vivo two-photon luminescence imaging of single gold nanorods Proc Natl Acad Sci U S A 2005 102 15752 15756 10.1073/pnas.0504892102 16239346 PMC1276057

[B23] Santamaria F Wils S De Schutter E Augustine GJ The diffusional properties of dendrites depend on the density of dendritic spines Eur J Neurosci 2011 34 561 568 10.1111/j.1460-9568.2011.07785.x 21771115 PMC3156966

[B24] Santamaria F Wils S De Schutter E Augustine GJ Anomalous diffusion in Purkinje cell dendrites caused by spines Neuron 2006 52 635 648 10.1016/j.neuron.2006.10.025 17114048 PMC1994115

