# Supplementary material for: Transient extracellular application of gold nanostars increases hippocampal neuronal activity
Source: J Nanobiotechnology. 2014 Aug 20;12:31. doi: 10.1186/s12951-014-0031-y (PMC4422288; doi:10.1186/s12951-014-0031-y)
Supplement: Additional file 1: — “Analysis of fluorescence correlation spectroscopy (FCS) measurements of star shaped nanoparticles”. The figure contains two panels showing FCS measurements to determine the concentration of gold nanoparticles in solution and a comparison with theoretical calculations. [file s12951-014-0031-y-S1.pdf]

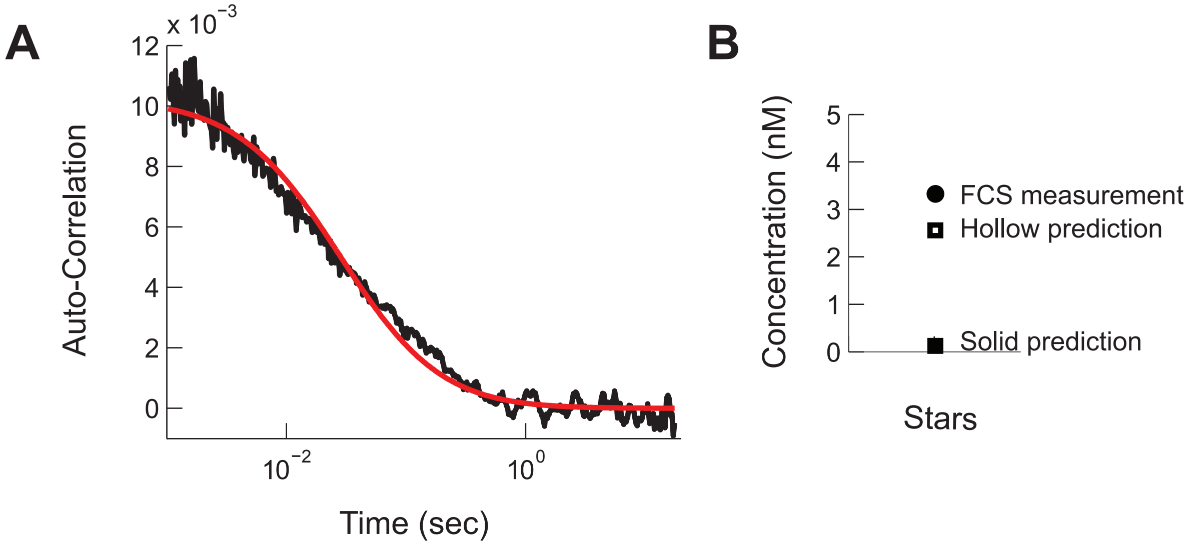

Supplementary Material Figure 1. Analysis of fluorescence correlation spectroscopy (FCS) measurements of star shaped nanoparticles. (**A**) Experimentally obtained auto-correlation curves (black) fitted with the FCS function (red). (**B**) Comparison of gold nanoparticle concentration extracted from A (solid circle) and predicted concentration based on chemical formulation and average shape assuming a hollow (hollow square) or solid (filled square) nanoparticle.
